# Molecular Detection and Identification of Piroplasm in Cattle from Kathmandu Valley, Nepal

**DOI:** 10.3390/pathogens12081045

**Published:** 2023-08-15

**Authors:** Medhavi Dhakal, Tulsi Ram Gompo, Prakash Devkota, Sharmila Chapagain Kafle, Janak Raj Subedi, Haiyan Gong, Hiroaki Arima, Richard Culleton, Masahito Asada, Kishor Pandey

**Affiliations:** 1 Central Department of Zoology, Institute of Science and Technology, Tribhuvan University, Kathmandu 44601, Nepal; medhavidhakal514@gmail.com (M.D.); janzoology@gmail.com (J.R.S.); 2 Central Veterinary Laboratory, Kathmandu 44600, Nepal; tulsigompo@gmail.com (T.R.G.); devkota2000@gmail.com (P.D.); sharmilakafle2@gmail.com (S.C.K.); 3 Shanghai Veterinary Research Institute, Chinese Academy of Agricultural Sciences, Shanghai 200241, China; gonghaiyan@shvri.ac.cn; 4 Department of International Health and Medical Anthropology, Institute of Tropical Medicine, Nagasaki University, Nagasaki 852-8523, Japan; h.arima@nagasaki-u.ac.jp; 5 Division of Molecular Parasitology, Proteo-Science Centre, Ehime University, Ehime, Matsuyama 791-0295, Japan; culleton.richard.oe@ehime-u.ac.jp; 6 Research Unit for Global Infection Control, National Research Center for Protozoan Diseases, Obihiro University of Agriculture and Veterinary Medicine, Obihiro 080-8555, Japan; masada@obihiro.ac.jp

**Keywords:** *Babesia*, *Theileria*, cattle, PCR, Nepal

## Abstract

Background: Tick-borne protozoan parasites (TBPPs) cause significant problems for domestic animals’ health in Nepal. TBPPs are routinely diagnosed by labor-intensive blood smear microscopy. In Nepal, there are some reports of *Babesia* and *Theileria* in cattle, although species identification is rarely performed. Therefore, we performed conventional nested PCR (nPCR) followed by sequence analysis to identify TBPP species infecting cattle in Nepal. Methods: One hundred and six blood samples were collected from cattle in the Kathmandu Valley. Thin blood smears were prepared for microscopic examination. Parasite DNA was extracted from the blood, and nPCR and sequencing were performed to identify the TBPPs present. Results: Among the 106 samples, 45 (42.5%) were positive for piroplasm (*Babesia* spp. and *Theileria* spp.) via microscope observation and 56 (52.8%) samples were positive via nPCR. The obtained PCR products were used for direct sequencing, and we identified the species as *B. bigemina*, *B. bovis*, *T. annulate* and *T. orientalis*. Phylogenetic analyses showed that the *B. bovis*, *B. bigemina* and *T. orientalis* sequences from this study belonged to each species clade. On the other hand, *T. annulate* was divided into two clades in the analysis, and our *T. annulate* sequences were also divided in these two clades. The piroplasm-positive cattle showed lower hemoglobin and red blood cells than healthy cattle. Conclusions: To the best of our knowledge, this study is the first to apply molecular detection and species determination of TBPPs in cattle in Nepal. The results of this study may be used as a starting point for the development of successful TBPP surveillance and prevention programs in Nepal.

## 1. Introduction

Livestock farming is an integral component of the Nepalese household economy and contributes 34% of the agricultural gross domestic product (GDP) [1]. The most important livestock products include milk (62.6%), and meat (32.4%) [2]. Cattle and buffalo make up approximately 80% of the total livestock in the nation [2]. However, the livestock sector suffers from a number of diseases caused by bacteria, viruses, and parasites [3]. Among the parasitological problems, the damage caused by tick-borne protozoan parasites (TBPPs) is considered very high [4]. The prevalence of TBPPs is one of the major causes of the reduced productivity of livestock, and it causes substantial economic loss to livestock industries [5]. For instance, TBPPs are responsible for severe economic losses due to pathogen control costs, along with a decrease in body weight and milk production [6]. 

In Nepal, cattle are raised only for milk production and not for slaughter and meat production by law, as they are worshipped by the Hindu people as earthly incarnations of the goddess Lakshmi. Nepal had 7.4 million cattle and their population has increased by 3% in the past 10 years [7]. Ticks, one of the many ectoparasites, have a significant negative impact on Nepal’s cattle industry [4,5]. Despite a large population of cattle with ticks, which are known vectors of infectious diseases, the diseases they transmit remain uncharacterized in Nepal. 

The piroplasms *Babesia* and *Theileria* are major tick-transmitted protozoa that infect cattle [8]. Piroplasms are intra-erythrocytic parasites and cause severe disease and death in infected animals [9]. The major causative agents of babesiosis in cattle are *Babesia bovis*, *Babesia bigemina*, *Babesia divergens*, and *Babesia major* [10]. *Babesia*, transmitted by hard ticks, is trans-generationally transmitted in ticks via transovarial passage to eggs [11]. Theileriosis in cattle is caused by *Theileria annulata* and *Theileria parva*. It is an intracellular protozoan parasite that infects both leukocytes and erythrocytes in cattle. Mortality rates can be reduced with early diagnosis and proper treatment. 

TBPPs can cause high mortality and morbidity in livestock if not accurately diagnosed and quickly treated. Currently, in Nepal, TBPPs are diagnosed by light microscopy of blood smears, a technique which can often miss low-level infections. Molecular detection of *Babesia* spp. and *Theileria* spp., however, enables identification of the TBPP species in the blood of cattle before the appearance of clinical signs. The prevalence of *Babesia* and *Theileria* has been reported in Nepal following microscopic examination, although species identification has never been carried out. Here, we report the results of a survey in which nested PCR (nPCR) and sequencing analysis were performed to determine the TBPP species in cattle from the Kathmandu Valley, Nepal.

The current study aims to produce baseline data for piroplasm identification in cattle in the Kathmandu Valley using conventional molecular techniques and to record hematological changes in infected animals. Additionally, the phylogenetic relationship of the isolates infecting cattle was examined.

## 2. Materials and Methods

### 2.1. Study Area

Nepal is a landlocked country bordering India to the east, west, and south and China to the north. Nepal can be topographically divided into three regions; mountain, hill, and terai. About 66% of the population are engaged in agriculture [12]. The Kathmandu Valley, home to the capital city of Kathmandu, is located in the Bagmati province of Nepal and is surrounded by four mountain ranges: the Shivapuri hills, Phulchoki, Nagarjun, and Chandragiri. The valley is made up of three districts: Kathmandu district, Lalitpur district, and Bhaktapur district. The valley is 665 square kilometers in size and is located on average 1350 m above the mean sea level [13]. The Kathmandu Valley had 104,141 head of cattle (Kathmandu = 51,357, Lalitpur = 23,736, and Bhaktapur = 29,048) [7].

### 2.2. Blood Sample Collection

**Figure 1:**
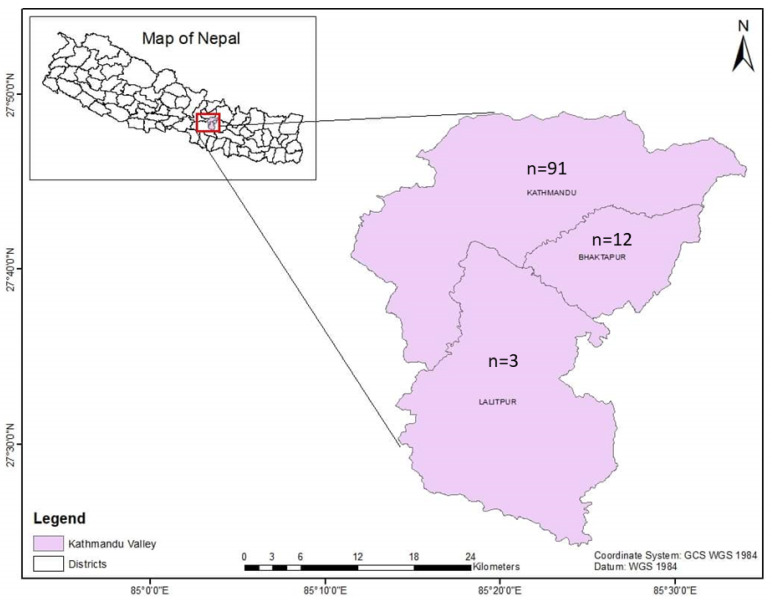
Map of Kathmandu Valley showing the origins of the samples.

The Central Referral Veterinary Hospital and Central Veterinary Laboratory are located in the Kathmandu district. In this veterinary hospital-based study, a total of 106 blood samples from cattle were received from the three districts of the Kathmandu Valley (Kathmandu (*n* = 91); Lalitpur (*n* = 3); Bhaktapur (*n* = 12)) (Figure 1) from March to November 2022. The samples were collected during summer (*n* = 53) and the rainy season (*n* = 53). Blood samples from the cattle that had fever and loss of appetite were submitted to the Central Veterinary Laboratory, Tripureshwor, Kathmandu, from the Central Referral Veterinary Hospital for a proper diagnosis of blood-borne infections. Blood samples were collected by puncturing the jugular veins of the cattle, and the samples were kept in EDTA tubes for hematological, microscopic, and molecular analysis. All the samples were collected by an experienced veterinarian, and efforts were made to limit the pain to the animals as much as possible during the blood collection. 

### 2.3. Microscopic Examination

Thin blood smears were prepared, air-dried, fixed with methanol, and stained with Giemsa’s solution. The stained slide smears were examined via microscopy for the presence of parasites under 100× oil immersion objective lens magnification (Optika Srl, Ponteranica, Italy). The presence of a single piroplasm was considered a positive case, and a minimum of 5000 RBCs were screened before a sample was considered negative.

### 2.4. Molecular Analysis

#### 2.4.1. DNA Extraction

The blood samples were stored at −20 °C until DNA extraction. DNA was extracted using a commercial DNA extraction kit (QIAamp^@^ DNA Blood Mini Kit, QIAGEN, Hilden, Germany) according to the manufacturer’s protocol. The DNA was eluted in 50 µL elution buffer. The extracted genomic DNA was kept at −20 °C for further molecular analysis. 

#### 2.4.2. Nested PCR 

The genomic DNA samples from the cattle were analyzed via nPCR using previously used methods [14]. The 18S ribosomal RNA gene of the parasite was amplified by means of PCR to detect *Babesia* and *Theileria* parasites utilizing BTH18S-1 primers for the primary amplification, followed by nPCR using BTH18S-2 primers. First, the PCR was performed in a 25 µL volume containing 12.5 µL Emerald Taq (Takara, Japan), 0.5 mM of each primer (BTH 18S-1F (GTGAAACTGCGAATGGCTCATTAC) and BTH 18S-1R (AAGTGATAAGGTTCACACAAAACTTCCC)), and 1 µL of DNA template. The thermocycler conditions for the PCR amplification were as follows: initial denaturation at 95 °C for 3 min, followed by 40 cycles (denaturation at 95 °C for 30 s, annealing at 55 °C for 30 s and elongation at 68 °C for 90 s) and final extension at 68 °C for 5 min using a Peltier Thermal Cycler (MyGene^TM^ L Series). The first PCR products were diluted five times and used for the nPCR. The second PCR (nPCR) amplification was performed under the same thermocycling conditions as the first PCR, except using 0.2 µL of each primer (BTH 18S-2F (GGCTCATTACAACAGTTATAGTTTATTTG) and BTH 18S-2R (CGGTCCGAATAATTCACCGGAT)). The amplified second PCR products were subjected to 1.5% agarose gel electrophoresis, stained with ethidium bromide, and then visualized under a Clear View UV Transilluminator.

#### 2.4.3. Sequencing

To identify the piroplasm, nine randomly selected nPCR-positive samples were sequenced. The sequence analysis was performed according to a previously described method [15]. Briefly, the second PCR products were treated with 2 µL of SAP-Exo (Jena Bioscience, Jena, Germany) for 5 µL of the PCR product, which was then incubated in a thermocycler at 37 °C for 10 min and 80 °C for 10 min. Following the manufacturer’s instructions, the purified products were used in a sequencing reaction with a BigDye^TM^ Terminator v3.1 Cycle Sequencing Kit (Applied Biosystems, Foster city, CA, USA) utilizing BTH 18S primers. These sequencing reactions were purified and analyzed using an automatic 3500XL Genetic Analyzer (Applied Biosystems, CA, USA). The sequences were cut at both the 5′ and 3′ ends before analysis using BioEdit (https://bioedit.software.informer.com/7.2/, accessed on 1 May 2023). Nucleotide BLAST was used to compare the acquired sequences to those found in public databases on the NCBI website (http://blast.ncbi.nlm.nih.gov/Blast.cgi, accessed on 1 May 2023). The obtained sequences have been deposited in the International Nucleotide Sequence Database via the DNA Data Bank of Japan (with the accession numbers LC762262–LC762269). 

### 2.5. Phylogenetic Analysis

The sequences were aligned and phylogenetic trees were constructed using the maximum likelihood method. Since LC762262 and LC762265 were aligned in one group, and LC762266 to LC762269 were also aligned in the other group, these sequences were analyzed independently. LC762263 and LC 762264 were shorter than 500 bp and not used for this analysis. The sequence data obtained from Piroplasm DB (Release 62, https://piroplasmadb.org/piro/app, accessed on 1 May 2023) and several sequences obtained from the BLAST results were aligned and indels or undetermined nucleotides were excluded using BioEdit. The substitution model selection and phylogenetic analysis were performed using IQ-Tree ver. 1.6.12 (http://www.iqtree.org/, accessed on 1 May 2023). The TIM3e+I+G4 (sequences including LC762262 and LC762265) or TIM2e+I+G4 (sequences including LC762266 to LC762269) model was selected and 1000 replicates of the ultrafast bootstrap analysis were performed. The phylogenetic trees were drawn using FigTree ver. 1.4.4 (http://tree.bio.ed.ac.uk/software/figtree/, accessed on 1 May 2023).

### 2.6. Hematological Analysis

The total white blood cell (WBC), red blood cell (RBC), and hemoglobin (Hb) concentrations were estimated using an automated hematology analyzer. Sahli’s hemoglobinometer was used to estimate the Hb, and a hemocytometer was used to calculate the combined RBC and WBC count.

### 2.7. Statistical Analysis

The results were plotted using GraphPad software after being tabulated in Microsoft Excel using the microscopic observation data for each sample. The inter-test agreements among the microscopy and PCR tests were calculated using the Kappa statistic [16].

## 3. Results

### 3.1. Microscopic Analysis 

**Figure 2:**
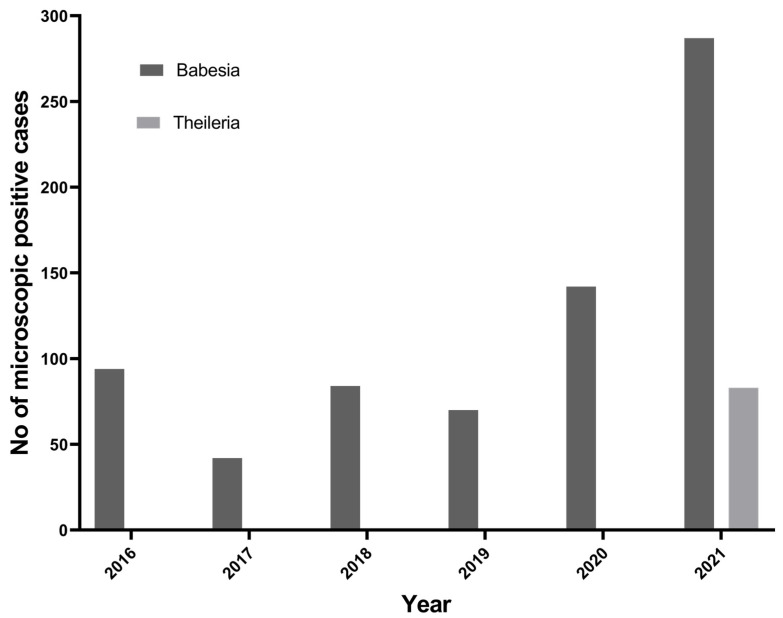
Trend of *Babesia* spp. and *Theileria* spp. reported in Kathmandu Valley.

To understand the trend of tick-borne protozoan parasites over the past 6 years (2016 to 2021) in the Kathmandu Valley, we plotted the number of microscopically positive TBPP cases from the data obtained from the Central Veterinary Laboratory, Kathmandu. In the past 6 years, there has been an increasing trend of *Babesia* spp. in Nepal (Figure 2). *Theileria* spp. cases in cattle have been reported since 2021.

From the Kathmandu Valley, 106 blood samples were taken from clinically sick cattle, and they were examined via microscopy from blood smears and used for the nPCR assay. The microscopic analysis revealed that 42.5% (45/106) of the blood smears were positive for piroplasm. 

### 3.2. Molecular Analysis

**Figure 3:**
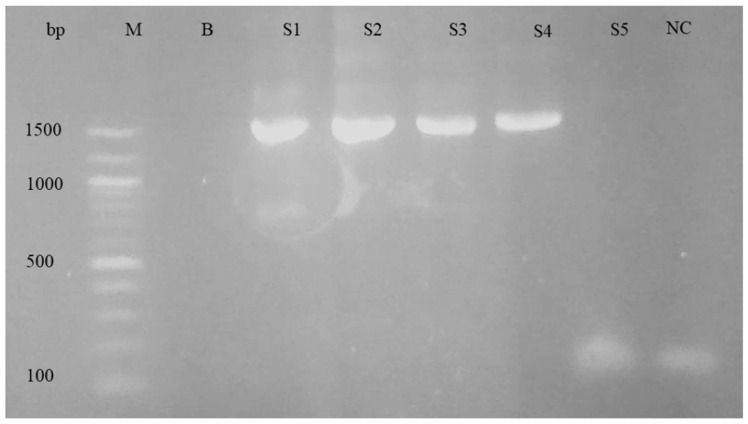
Agarose gel electrophoresis of nPCR products using 18s rRNA primers for piroplasm. Left to right: M, DNA ladder; B, blank; S1–S5, samples; NC, negative control.

For the purpose of differentiating the *Babesia* and *Theileria* species, nPCR and sequencing were performed. The nPCR was carried out using primers specific to the 18S rRNA genes of *Babesia* spp. and *Theileria* spp. When electrophoresis was performed on a 1.5% agarose gel, the nPCR analysis revealed a PCR product of about 1500 base pairs. Figure 3 shows an example of an agarose gel electrophoresis image. 

The gel electrophoresis picture from the positive controls showed the anticipated 1500 bp band; however, it was missing from the negative controls. According to the PCR assay, 56.2% (56/106) of samples were determined to be positive for piroplasm (Table 1). There was a fair kappa agreement between the two tests: the microscopy test and PCR test for the detection of piroplasm species (κ = 0.31, 95%CI: 0.13–0.49, *p* < 0.001).

In Kathmandu, 54.9% (50/91) were positive, whereas in Bhaktapur and Lalitpur, 41.7% (5/12) and 33.3% (1/3) were positive for piroplasm via nPCR. Out of 106 samples, 53 samples were collected during summer and 53 samples were collected during the rainy season. The piroplasm was detected higher during the rainy season 32 (60.4%) than during summer 24 (45.3%) via nPCR, which is statically not significant (*p* = 0.658). 

The PCR products from nine samples were selected for direct sequencing. The sequences were compared using Nucleotide BLAST with those deposited in the public database. Among the nine sequences, one sequence showed a *T. annulata*-like sequence; however, the data showed several double peaks and we could not determine the sequence. The other eight sequences were determined and deposited in the database (LC762262–LC762269, Table 2). 

The sequence LC762262 was identical to several *T. orientalis* sequences (990/990, 100%). The sequence LC762263 showed high similarity to *T. orientalis* group sequences (427/428, 99.77%). The sequences LC762264, LC762267 and LC762268 showed high similarity or were identical to several *T. annulata* and *T. orientalis* sequences (462/464, 99.57%; 901/901, 100%; 901/901, 100%, respectively). The sequence LC762265 showed high similarity to a *B. bigemina* sequence (918/919, 99.89%). The sequence LC762266 was identical to several *T. annulata* sequences (918/918, 100%). The sequence LC762269 was identical to several *B. bovis* sequences (918/918, 100%). 

**Figure 4:**
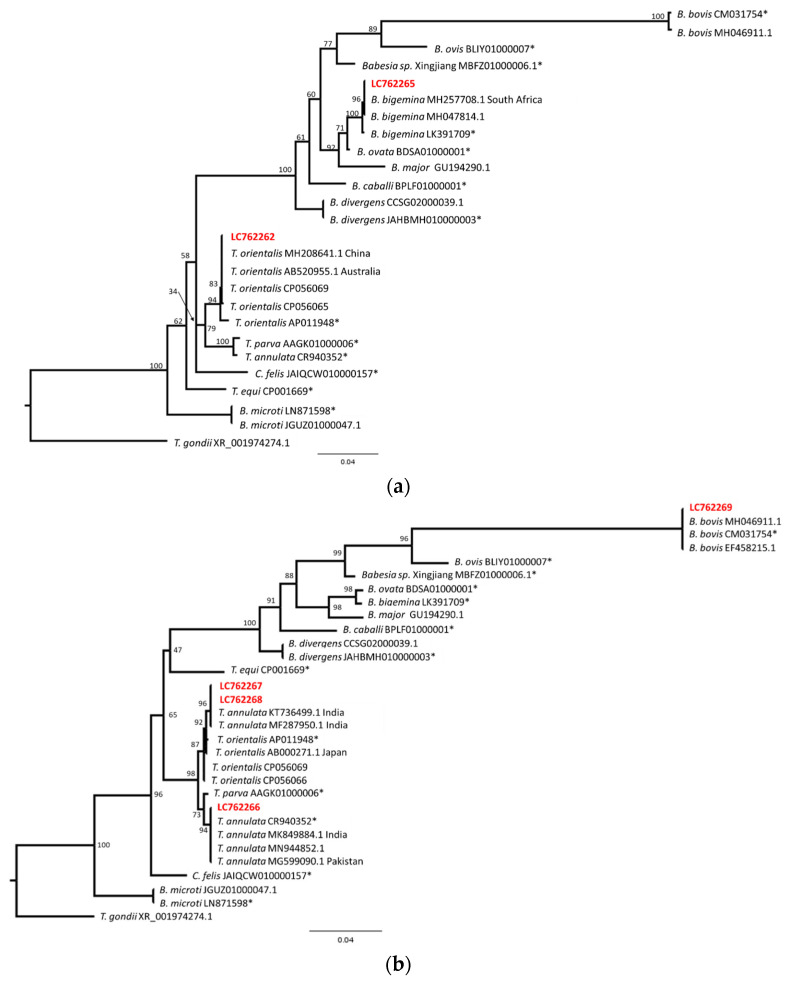
Phylogenetic relationship of piroplasm sequences from Nepalese cattle. The sequences were aligned and indels or undetermined nucleotides were excluded. (**a**) The resulting 858 bp (LC762262, LC762265) was used for the maximum likelihood analysis. The bootstrap values and length of the substitutions/site (0.04) are indicated. (**b**) The resulting 866 bp (LC762266–LC762269) sequence was used for the maximum likelihood analysis. The bootstrap values and length of the substitutions/site (0.04) are indicated. * Indicates reference genome sequences.

To obtain further insight into the obtained sequences, phylogenetical analyses were performed. The sequences LC762262 and LC762265, or LC762266 to LC762269, were aligned with several sequences from the PiroplasmaDB and BLAST search results and were analyzed via the maximum likelihood method. The resulting tree indicated that the LC762262 and *T. orientalis* sequences formed one clade, while the LC762263 and *B. bigemina* sequences formed another clade (Figure 4a). Another tree indicated that the LC762269 and *B. bovis* sequences formed one clade (Figure 4b). The major *Theileria* sequences separated into two clades with a high bootstrap value (98), and one clade contained *T. annulata* and *T. parva*. LC762266 was included in the *T. annulata* clade with the reference genome sequence and the other *T. annulata* sequences from India, Pakistan and other regions. On the other hand, another clade contained *T. orientalis* sequences and a few *T. annulata* sequences from India. The sequences LC762267 and LC762268 were located in the Indian *T. annulata* clade. This result suggests that LC762267 and LC762268 may be *T. annulata*; however, further sequence analysis and analysis of clinical manifestation will be necessary. Nevertheless, our sequence data clearly indicate that *B. bovis, B. bigemina*, *T. annulata* and *T. orientalis* are circulating in cattle in the Kathmandu Valley.

### 3.3. Hematological Analysis 

**Figure 5:**
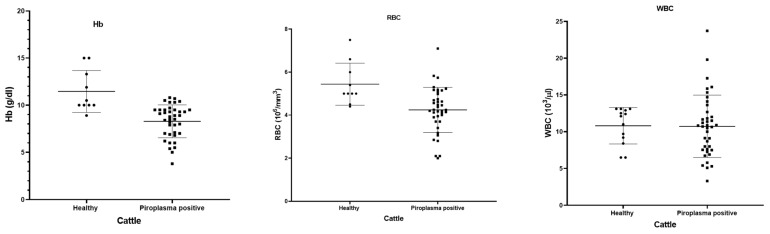
Hematological parameters (hemoglobin, RBC and WBC) shown by dot-plot for healthy (black round symbols) and piroplasm-positive (black box symbols) cattle.

Fever, decreased milk production, pale mucous membrane, and loss of appetite were the main clinical symptoms shown by the cattle. The hematological data of 42 microscopically piroplasm-positive cattle and 10 healthy cattle were compared (Figure 5). The piroplasm-positive cattle had significant lower Hb (*p* = 0.0001) and RBC counts (*p* = 0.0021) than the normal healthy animals, although there was no significant difference in the WBC between the healthy and piroplasm-positive cattle (*p* = 0.9505).

## 4. Discussion

Piroplasmosis is a tick-borne disease caused by protozoan parasites of the genera *Babesia* and *Theileria*, which infect the blood cells of cattle. This study is the first to identify *B. bovis*, *B. bigemina*, *T. annulata* and *T. orientalis* in cattle from Nepal using molecular diagnosis. 

In 1989, *Babesia* was detected in two cows in the Kathmandu Valley from microscopic examination that was imported from India [17]. After that, several studies reported the piroplasm in Nepal. In the present study, the positive cases of piroplasm were 42.5% according to the microscopic examination. A study conducted in the western terai region of Nepal found that the positive cases of piroplasm in cattle were between 10% and 19.3% [18,19,20]. Other studies conducted in different regions of Nepal found that the positive cases of piroplasm in cattle were higher than the present study: 72.2% (121/163) in the eastern part of Nepal (clinical diagnosis), between 50% and 75% [18,19,21,22,23,24]. In India, the prevalence of piroplasm ranges from 2% to 33%, depending on the region [25,26]. The present study has higher positive cases because it is a hospital-based study and the samples used for this study came from unhealthy cattle that were brought to the central veterinary laboratory for seeking diagnosis and treatment.

In the present study, 52.8% of samples were detected for piroplasm via nPCR. The molecular test (nPCR) is considered the most sensitive and specific test to detect the parasites, even in the case of the presence of low parasitemia [27,28,29]. We found several microscopically (13/45) positive samples were negative for the nPCR. This could be due to the microscopic examination being labor intensive and requiring skill to detect and identify parasites in RBC and being very easy to misdiagnosis [30]. Similarly, many microscopically negative samples were positive (24/61) via nPCR. PCR is a molecular technique that amplifies specific DNA sequences of the piroplasm parasite, making it easier to detect its presence in a blood sample. PCR offers several advantages over microscopic examination. It is highly sensitive, and it can detect even low levels of the parasite’s DNA [31,32]. In comparison to microscopy, PCR approaches have greater piroplasm detection rates, highlighting the importance of employing molecular tools to identify carrier hosts for efficient control efforts.

While analyzing the seasonal positive cases of *Babesia*, the higher positive cases were found during the rainy season compared to the summer season. The higher positive cases during the rainy time were likely because the climate is hot and humid, which is suitable for the breeding of the tick population. When the tick population increases, there is an increase in piroplasm cases in cattle [33]. 

In this paper, we present the first molecular evidence of *B. bovis* and *B. bigemina* infection in Nepalese cattle. Both *B. bovis* and *B. bigemina* have been reported in India [34,35,36]. Higher incidences of piroplasm have been reported in Sri Lanka, with an incidence rate of 73.1% via PCR and the most common piroplasm being *T. orientalis* (53.5%) and *B. bigemina* (30.1%) [37]. Both *T. annulata* and *T. orientalis* were found in the cattle blood in our current investigation. Similar findings have been reported in India and Pakistan [38,39,40,41]. 

Although the present work is not focused on the distribution of ticks, ticks, the vector for piroplasm, have been reported in different regions of Nepal, including Kathmandu Valley [5,42,43]. In the mid-western terai region and hilly region of Nepal, *Rhipicephalus microplus* is the most prevalent tick, followed by *Haemophysalis* spp., *Ixodes* spp., and *Amblyomma* spp. [4,5]. The Kathmandu Valley is located in Nepal’s central hill regions, where the temperature (above 25 during summer and the rainy season) and humidity are favorable for tick reproduction, survival and activity. This could be the reason for the higher positive cases reported in the present study. 

We performed a sequence analysis of a limited number of samples. We identified DNA from *B. bovis*, *B. bigemina*, *T. annulata* and *T. orientalis* in blood samples from cattle in the Kathmandu Valley. According to the phylogenetic tree incorporating the LC762265 sequences, the cattle-derived *B. bigemina* variation is remarkably similar to isolates from other parts of the world, indicating significant conservation at this locus [14,44]. *Babesia bovis* has previously been reported in India [30]. In the present study, two sequences showed *T. orientalis*, one sequence showed *T. annulata* and three sequences showed *T. annulata*/*orientalis*. Both *Theileria annulata* and *T. orientalis* have been reported in cattle in central, eastern and north-eastern India [30,45,46], Pakistan and Bangladesh [47]. The existence of various tick species may account for the variation in the distribution of the two *Theileria* species. The sequence of one sample was similar to that of *T. annulata*, although the sequence data contained multiple double peaks. The species could not be determined, which suggests the cattle were co-infected with different species. Co-infection of *Babesia* and *Theileria* species has been reported [48,49].

Piroplasmosis is a significant concern for livestock farmers, as it can cause reduced milk production, weight loss, and even death in affected animals. In the present study, anemia was more prevalent in the piroplasm-infected cases compared to the healthy animals. The lysis of RBCs caused by parasite proliferation, and the subsequent clearance by the reticuloendothelial system, all contribute to anemia [50]. Similarly, the constant blood loss caused by tick suck outs also contributes to anemia [51].

This study’s findings could be useful to provide strong evidence, especially in light of the significant economic harm, that piroplasm pose a serious problem to livestock farming in Nepal. The information gained in this study will be valuable as a starting point for developing efficient TBPP molecular surveillance and prevention strategies in Nepal.

This study had some limitations. In the present study, a selected number of samples were used for sequencing. We did not perform cloning of the sample that showed multiple peaks. Nevertheless, this study provided novel information about the presence of piroplasm in cattle in Nepal via a molecular method that will help animal stakeholders to control these diseases in the study areas.

## 5. Conclusions

Our results showed that PCR is better than microscopic examination for the accurate diagnosis of piroplasm. This study confirmed the presence of four piroplasm species, namely *B. bigemina*, *B. bovis*, *T. annulata* and *T. orientalis*, infecting cattle which had not been previously reported in Nepal using PCR. These findings provide fresh insights into piroplasm in Nepal and indicate that veterinarians treating cattle need to be aware of piroplasmosis.

## Figures and Tables

**Table 1:** Comparison between the nPCR and microscopical examination to make a diagnosis of piroplasm.

Microscopic	PCR		
	Positive (*n*)	Negative (*n*)	Total, *N* (%)
Positive	32	13	45 (42.5%)
Negative	24	17	61 (57.5%)
Total	56 (52.8%)	50 (47.2)	106

**Table 2:** Accession numbers and the results of the Nucleotide BLAST search.

	Accession No.	Sequence Length	Result of BLAST Search Species (Accession No, Identity (%))	Indicated Species
1	LC762262	990	*T. orientalis* (MH208641.1, AB520955.1 and others, 990/990 (100))	*T. orientalis*
2	LC762263	428	*T. orientalis/sergenti/buffeli* (MH208641.1, MT355456.1, MH327775.1 and others, 427/428 (99.77%))	*T. orientalis*
3	LC762264	464	*T. annulata* (MF287950.1 and others, 462/464 (99.57%)), *T. orientalis* (LC576818.1 and others, 462/464 (99.57%))	*T. annulata* or *T. orientalis*
4	LC762265	919	*B. bigemina* (MH047814.1, 918/919 (99.89%) followed by LC645217.1 and others 917/919 (99.78%))	*B. bigemina*
5	LC762266	918	*T. annulata* (MT341858.1, MG599090.1 and others, 918/918 (100))	*T. annulata*
6	LC762267	901	*T. annulata* (MF287950.1 and others, 901/901 (100%)), *T. orientalis* (LC576818.1 and others, 901/901 (100%))	*T. annulata* or *T. orientalis*
7	LC762268	901	*T. annulata* (MF287950.1 and others, 901/901 (100%)), *T. orientalis* (LC576818.1 and others, 901/901 (100%))	*T. annulata* or *T. orientalis*
8	LC762269	893	*B. bovis* (MH046911.1, EF458215.1 and others, 893/893 (100%))	*B. bovis*

## Data Availability

The datasets generated and/or analyzed during the current study are available in the manuscript.
